# Investing in an Olympic agenda: from Rio to Tokyo and beyond

**DOI:** 10.3389/fspor.2024.1507523

**Published:** 2025-01-07

**Authors:** Eduardo Russo, Ariane Roder Figueira, Leonardo Jose Mataruna-Dos-Santos

**Affiliations:** ^1^ Business School, Tecnologico de Monterrey, Mexico City, Mexico; ^2^ The COPPEAD Graduate School of Business, Federal University of Rio de Janeiro, Rio de Janeiro, Brazil; ^3^ School of Management, Canadian University Dubai, Dubai, United Arab Emirates

**Keywords:** Olympic Games, Olympic investments, Rio 2016, Tokyo 2020, investment sustainability

## Abstract

**Context:**

This study was inspired by the considerable risks and diminishing enthusiasm among societies to invest in Olympic agendas, which traditionally involve billions of dollars, various opportunities, and complexities for host countries.

**Purpose:**

The objective of this study was to evaluate the risks and benefits of long-term equity investments for companies and governments engaged in the Olympic movement.

**Method:**

Qualitative methodologies were employed for this research, utilizing a multi-case approach that included 38 comprehensive interviews with companies and entities impacted by the Rio 2016 and Tokyo 2020 Olympic Games.

**Results:**

Consequently, a theoretical framework titled “Risks and Opportunities Related to Olympic Investments” is presented to elucidate the dynamics of investment flows, competitive pressures in specific sectors, and future threats and trends for the Olympic movement.

**Findings:**

The research revealed that the gigantism of past editions exerted significant pressure on organizers and companies to adopt new management practices and enhance investment planning, striving for minimal environmental impact and long-term economic sustainability. These insights aid scholars, practitioners, and policymakers in making informed decisions about resource allocation in Olympic contexts, and highlight the necessity for updated strategic frameworks to ensure the viability and positive impact of future Olympic Games.

## 1 Introduction

Assuming that the considerably increasing cost of hosting the Olympic Games over the years has created a growing disinterest in potential hosting cities, countries, and societies, this research aims to analyze the risks and opportunities related to long-term equity investments for companies and governments engaged in an Olympic agenda. To this end, two Summer Olympic Games were analyzed: Rio in 2016 and Tokyo in 2021—this singular edition took place in the middle of one of the biggest pandemics in recent human history. As a timeframe for analysis, the focus was to observe the pre-event moment by monitoring the cities’ preparation for the BID^1^ and their legacy expectations.

Although Rio de Janeiro, Brazil, and Tokyo, Japan, are very distant examples for a possible comparison in terms of culture and institutions, both cities had clear problems with hosting their Olympics. While Tokyo had to negotiate with an Olympic postponement and a Games behind closed doors in the middle of a period of social isolation, in Rio de Janeiro, most of the problems seem to have occurred after the event had finished. Whether due to political and institutional instability or a health crisis, the issue is that there were additional complications for the companies and organizations that invested in both events. The tourism, hospitality, and services sectors were the most affected. Historically, mega-events have significantly impacted these businesses due to the increase in tourist flow (1). Consequently, many organizations invest substantial resources during these cycles (2).

However, if well-planned, mega-events can provide several benefits for the hosting regions (3). Milan (4) presented successful examples of past host cities such as Barcelona in Spain (1992), Atlanta in the United States (1996), and Sydney in Australia (2000). According to the Milan (4), all those cities experienced a large contribution of investments which was able to bring accelerated economic growth to the locations most affected by the Games, exponentially reducing the unemployment rates and positively transforming the lifestyles of the local population.

In contrast, we have the case of the Athens Games in 2004. Despite being important culturally due to Greece being the nation where the Olympic spirit had begun, the Greek government announced that the final cost of the project was almost double the initial estimated budget, in addition to having experienced debt increase and shrinking GDP in both the pre-and post-Olympic periods (5). The 2004 Games were also marked as the beginning of the Greek economic crisis, where in 2010 the state's debt reached 144.9% of GDP, compared to 77% in the 2000s (6).

Even with the positive and negative aspects of hosting an Olympics, it is common to have great expectations immediately after the Olympic nomination. There is the possibility of socioeconomic legacies that could directly affect the lives of citizens facilitated by the investments that mega-events could bring to hosting regions, with the transformation of urban mobility, tourism, culture, infrastructure, and sports in the city (7). But for this to be possible, it is first necessary for companies and governments to be willing to commit to large (billions of dollars) long-term equity investments in the local market (8), the value of which has been considerably increasing over the years.

Tangible and intangible legacies have been investigated by different researchers over the past few years. However, their results have limitations because of the difficulty in determining the appropriate time frame—where does the Olympic legacy begin or end? (9, 10). Despite legacy being one of the pillars of an Olympic candidacy required by the International Olympic Committee (IOC), after the end of the event, attention is turned to the next edition, leaving the past hosting locations to manage it alone (11). Therefore, it is relevant to reflect on the legacy that the investment from international mega sporting events could bring to the hosting locations since the positive or negative impacts brought by these will contribute to the sustainability of the Olympic Games model in the medium and long term.

## 2 Literature review

### 2.1 The growing complexity of the Olympic Games

In order to discuss Olympic investment, we must first reflect on the governance of an Olympics, that is, who they are and how the institutions responsible for defining not only the investment rules but also other policies, actions, and processes of all phases of the event are structured (12). In this sense, the decision to invest in a mega sporting event takes into consideration not only an agent, but an entire network, where the level of control, stability, and coordination of the leaders will be decisive for the achievement of objectives and the consequent success of the event (13). However, Prüschenk (14) explains that due to the complexity and extension of the network, a decentralization of power and decision over investments is the most common, where most decisions are taken in a participatory manner. Thus, it is common to observe tensions between different actors in complex situations where there is not always a convergence of interests, especially if we consider local and international entities.

Due to the increasing complexity of the Games since the 1990s, driven mainly by the professionalization of sport and the growth in the number of stakeholders, thinking about governance over Olympic investments has made more and more sense (15). In one of the most recent works, Chappelet (16) portrays the complexity of this network and maps the main actors that, besides the International Olympic Committee, may interfere in decisions about the event. These include the local organizing committee, national Olympic committees, governments, sponsors, regulatory agencies, international and national sports federations, the media, athletes, and delegations, in addition to spectators. Although the latter do not have much influence over the event's investments, they end up being capable of putting great pressure on the main entities responsible for it. Among so many actors, Sorensen (17) argues that the only way to achieve a positive outcome is through the implementation of meta-governance mechanisms where formal procedures have been established, with the creation of goals and quality and control standards, in addition to a strong and participative decision mechanism.

As a way to guide these decisions, Considine and Afzal (13) draw attention to the fact that despite the challenges presented by many Olympic projects, the IOC shares some principles to be followed by those responsible for delivering the event, namely, transparency (clear rules and communication, in addition to the publication of results), democracy (decisions in a participatory manner), responsibility (clear and well-defined tasks for each of the actors), autonomy (of the host city), and social responsibility (ensuring the legacy for the environment and society). To operationalize these guidelines, Parent (18) highlights the importance of the figure of the local organizing committee, which acts in the coordination of efforts from the planning phase since the BID, during the event itself, and even some period after the end of the Games, in the intention of demobilizing the structure and deliver the legacy plan. Building on these ideas, Zintz and Gérard (19) emphasize the need for clear governance structures and accountability mechanisms to ensure the effective implementation of these principles.

Although the organizing committees have had different configurations through the Games editions, sometimes more independent and sometimes more subordinate to the local government, their mission has become increasingly complex precisely due to the gigantism of the most recent Olympic editions (15, 20). Since the first edition in 1896, the Games has grown from 14 events, 250 athletes, and 14 countries to 306 events, 11,238 athletes, and 206 countries in 2016. This trend continued with the inclusion of five new sports modalities in the Tokyo Games, increasing the number of events to 339 (21). In Paris (2024), a total of 38 modalities could be seen—a number considerably higher than the 21 practiced in the Games of the 1970s and 1980s. Thus, the size of the Olympic Games increased the network complexity level and consequent investment cost of the project, going against the sustainability and social responsibility pillars defended by the Olympic Agenda 2020 (22).

The increasing complexity of the Olympic Games has made the high standards required by the International Olympic Committee during the planning phase challenging, especially regarding the sustainability of the project. This requirement, fulfilled by the host cities at the time of their candidacy, is one of the three pillars that must guide the next Olympic editions and is highlighted in the Olympic Agenda 2020 and in its update, Olympic Agenda 2020 + 5 (23). Despite this requirement having emerged in the 1990s amid the increased representation of the sustainable development agenda at the UN, after approximately three decades, it is still a topic that divides opinions, since in practice it has been difficult to enact measures that make the mega sporting events on the planet truly sustainable in its broadest sense (24), which takes into account not only ecological aspects but also considers social and economic impacts (25).

Despite the difficulty in quantifying the tangible, intangible, direct, and indirect costs of hosting an Olympics, over the last century, it was already possible to observe an increase in expense (26, 27). If at the Athens Games (1896) there was an expenditure per athlete of 17,000 dollars in regressive values, at the Berlin Games (1936) this amount was already 53,000 dollars, reaching the mark of USD 1 million in the first Asian Games in Tokyo in 1964. The Barcelona Games (1992), considered a general success, was also the most expensive edition of the 20th century at USD 7 billion, almost double the USD 3.6 billion spent by Atlanta in 1996 (28). The escalation in expenditure will only increase in subsequent editions. These high values, becoming more common in events of this size, may open discussion about the real sustainability of the Olympic projects (29).

### 2.2 The necessity for a more sustainable model for the Olympic Games

While sustainability should be a rule, in practice, it can be observed in the last 30 years that there have been isolated sustainability initiatives such as those in the Albertville (1992), Salt Lake City (2002), and Vancouver (2010) Games. The latter was one of the only editions to have completed its Olympic impact study, which ended up being abandoned years later in 2017 due to the cost involved (29). This lack of recent examples capable of proving to cities around the world that good Olympic planning can materialize in a sustainable Games model has been widely discussed in the IOC sphere and has driven away the interest of cities around the world in hosting a Games (30). This becomes clear with the withdrawal of the candidacies of Krakow and Stockholm from the 2022 Winter Games, and Budapest, Hamburg, and Rome from the 2024 Summer Games. With only Los Angeles and Paris remaining, the alternative ended up being to distribute the Games of 2024 and 2028 between these interested parties. This model was also followed with Brisbane's sole application for the 2032 Olympics.

Another problem presented in almost all Olympic projects, once again harming the sustainability of the model, has been the much higher expenditure than planned (31). Flyvbjerg et al. (32) pointed out that between the 1960s and 2016, there was an average expenditure that was 156% over budget with a negative contrast for the Sochi 2014 (+289%), Lake Placid 1980 (+324%), and Montreal 1976 (+720%) Games, making the Olympic Games a high-risk investment for both companies and governments. In this sense, many authors have questioned the real advantages of hosting mega sporting events (33), as, in some cases, instead of contributing to the sustainable development of the host cities, these events ended up generating an increase in public debt and negative economic impacts in the medium term.

Past Olympic editions have shown us that despite the economic logic that does not make clear the advantages of hosting the event, the possibility of long-term intangible legacies may be one of the main motivators that still leads to the search for locations to host mega-events in general (1). In this sense, it is necessary to first conceptualize this term which is often used in contexts that involve the Olympics. Cashman (34) understands the Olympic legacy in a large sense, encompassing issues beyond economics but also affecting the urban spaces, environment, education, politics, culture, sport, and memory of the hosting regions. Preuss (35), on the other hand, goes further by visualizing legacy in three distinct dimensions which can be planned or unplanned, positive or negative, tangible, and intangible. By this logic, the same event can leave positive legacies for some sectors, such as the tourism industry, and negative for others, such as the environmental impact.

Although legacy is currently an important part of the Olympic bid, the way it is later managed by the organizing committee can still be criticized (36). According to Lopes dos Santos and Gonçalves (37), in the years of preparation, the greatest concern seems to be with the delivery of the event and not with the impact that it can generate in the medium or long term. This is reflected by the fact that the local organizing committee is usually dissolved within 2 years after the Games while its positive or negative impacts may remain for decades. The Global Olympics Impact Report (OGGI), such as the one implemented by Vancouver in 2010, also follows this logic. Despite gathering information and generating relevant statistics that serve as a base for future editions, it does not have practical implications for incomplete legacy promises (38).

Regarding the assessment of the Olympic legacy from a tangible point of view, this topic has proved to be even more problematic for the winter editions (39). According to the authors, this has occurred because the structures developed for this type of event are even more difficult to convert for the benefit of the local population when compared to the Summer Olympic Games. To contribute to this panorama, Scheu et al. (40) pointed out that although the legacy theme has gained more relevance in recent years, the efforts of the cities are still very focused on the legacies that contemplate urban transformations as they are visible to the population and provide better justifications for the high expenses involved. In this sense, Li and McCabe (41) indicate that measurement of the intangible legacies of the Olympic Games becomes even more difficult due to the lack of indicators or studies. Thus, assessing the positive and negative consequences of mega sporting events for the image, culture, and local memory, has become a challenge for hosting locations (42).

## 3 Method

This research analyzed the risks and opportunities related to long-term equity investments for companies and governments engaged in an Olympic agenda. To do so, the Summer Olympic Games in Rio in 2016 and Tokyo in 2021 were analyzed—the latter was a singular edition that took place in the middle of one of the biggest pandemics in recent human history. To fulfill this objective, the present research was conducted using the case study method with a multiple-case approach following the protocol proposed by Eisenhardt (43). According to Yin (44), this method is widely used when circumstances are complex and can change when the solutions for those conditions have not been found before, when situations are highly politicized, and when there are many stakeholders.

In the case of Rio de Janeiro, seven different companies that at the time of the 2016 Olympics had representative activities in the city were interviewed. The companies, composed of both local and foreign groups, were chosen because they had, to a lesser or greater extent, expanded their equity investments in the city of Rio de Janeiro during the years that preceded the 2016 Olympic games. To better understand the movements these companies made in the period, another group of public agencies also received attention to this research. This was important to be able to triangulate the information received from the interviewees of the first group, totaling 17 in-depth, semi-structured interviews. Data collection took place through secondary sources from the analysis of scientific and journalistic articles and marketing material provided by some of the companies and entities visited, from primary sources through semi-structured interviews with managers of the local companies and people involved in the public life of the city of Rio de Janeiro. The conversations with the interviewees were transcribed, generating 238 pages of primary data that went through a coding process to categorize their responses.

The case of Tokyo unified the perspective of 21 interviewees belonging to 18 different entities who at the time of the postponement of the Tokyo Olympic Games in March 2020 were directly or indirectly affected by the decision. This combined perspective helped us identify the singularities of the Tokyo project and its alignment with the new sustainability standards brought by Agenda 2020, and the difficulties and adaptations that companies and organizers had to make amid the first Olympic postponement in history. Data collection took place through secondary sources of analysis such as scientific and journalistic articles. The primary sources were in-depth, semi-structured interviews with the Tokyo 2020 organizers, with middle and senior management of companies or entities selected, and with senior researchers from institutions around the world that followed the paths and changes in the Olympic movement. The transcription of the interviews generated an additional 248 pages of primary content that was later classified and categorized. The complete list of interviews can be found in Table 1. To guarantee confidentiality and anonymity, all the individuals’ names and positions have been omitted.

**Table 1:** Interviews.

No.	Organization	Pages transcribed	Time
Rio case			
R01	Accor Hotels	21	59 min
R02	Best Western Hotels & Resorts	15	42 min
R03	Best Western Hotels & Resorts	17	47 min
R04	Golden Tulip Hotels	17	66 min
R05	Marriott Hotels	04	Email
R06	Othon Hotels	08	53 min
R07	Venit + Mio Hotel	14	43 min
R08	Windsor Hotels	17	47 min
R09	Brazilian Hotels Association	16	61 min
R10	Federation of Industries of the State of Rio de Janeiro	18	59 min
R11	Rio 2016 Organizing Committee	18	50 min
R12	Rio Convention and Visitors Bureau	19	62 min
R13	Rio de Janeiro City Hall	17	55 min
R14	Rio de Janeiro City Hall	11	67 min
R15	Rio de Janeiro City Hall	15	57 min
R16	Rio Investment Agency	23	88 min
R17	Secretary of State for Sport, Leisure and Youth	04	Email
Tokyo case			
R18	London University of Arts	11	46 min
R19	Gestamp Autotech Japan	4	Email
R20	Sport & Society Research Network	11	44 min
R21	International Olympic Committee	5	Email
R22	UNESCO	23	86 min
R23	Canada Journal Exchange Programs	4	Email
R24	Nomura Securities Japan	4	Email
R25	Kraft Heinz North East Asia	4	Email
R26	Brazilian Judo Confederation	4	Email
R27	Hirata Corporation Japan	4	Email
R28	International Olympic Committee	29	104 min
R29	International Olympic Academy	20	69 min
R30	Federal University of Rio Grande	17	54 min
R31	American University in the Emirates	12	50 min
R32	AG Sports Consulting	24	85 min
R33	PwC Japan	5	Email
R34	Tokyo 2020 Organizing Committee	17	68 min
R35	Tokyo 2020 Organizing Committee	13	59 min
R36	Tokyo 2020 Organizing Committee	11	37 min
R37	International Military Sports Council	20	61 min
R38	Brazil Embassy in Tokyo	6	Email
Total		486	1,619 min

Source: prepared by the authors.

**Figure 1:**
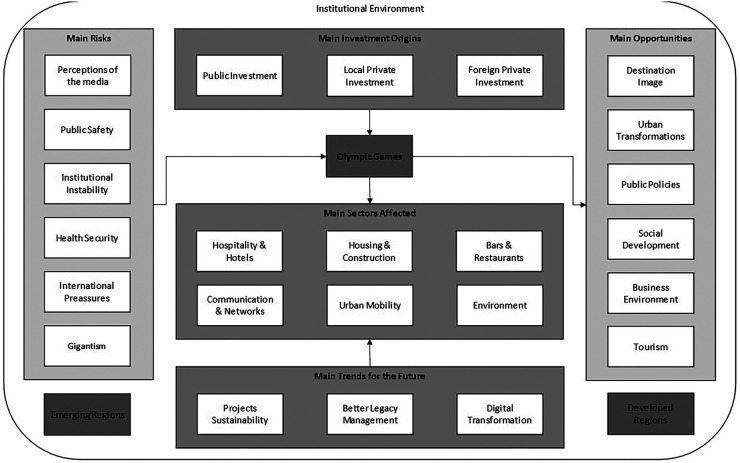
Risks and opportunities related to Olympic investments.

The field interviews were responsible for generating a rich collection of primary data, which, after analysis and categorization, enabled the identification of several variables that were divided into different groups. The coding process was conducted manually by the authors with the help of MS Excel software, who after identifying common points cited in the speech of two or more respondents, highlighted 24 variables that are presented in Figure 1. This dynamic helped us understand how specific sectors of the industry behave in terms of organic growth and investments during Olympic cycles.

As the information presented in this study was derived from interviews with various participants, it is important to underscore that the data may be influenced by the individual perceptions of those interviewed. This inherent subjectivity can introduce biases typically associated with qualitative research methodologies. Therefore, it is crucial to consider these potential biases when interpreting the findings, as they reflect the personal experiences and viewpoints of the participants rather than objective measures (45). According to Ravitch and Carl (46), qualitative research methods, despite their inherent biases, are particularly effective in exploring complex social phenomena as they allow for a deeper understanding of the context-specific dynamics and the subjective experiences of individuals. Nonetheless, this method remains relevant to the context being studied, as it provides valuable insights into the nuanced and complex experiences of stakeholders involved in Olympic investments.

## 4 Results and discussion

Even though 6 years separate the moment of the choice of city and the event of the Olympic Games, it is known that there is a lot of work required during this period to deliver an event within the minimum standards required by the IOC in terms of structures and legacies (10, 27, 35). At the time of their nomination, the ability of the Brazilian and Japanese agencies to deliver not only the venues but also all the infrastructure responsible for hosting the events was completely different, but in both cases, problems and difficulties arose in the management of the megaprojects. One of the Brazilian interviewees recalled that “In our case, political and economic instability is always a huge challenge, often causing delays and budget overruns that are difficult to manage.” If the lack of experience and an unstable political and institutional environment were two of the main complicating factors in the Brazilian case, Tokyo had to deal with COVID-19—one of the biggest pandemics in human history that completely changed the way the Olympic Games was planned and delivered (47). “The pandemic brought an unprecedented context in the recent history of the Olympic Games. At that moment, none of us from the local organizing committee or even the IOC knew what to do,” said one of the Japanese interviewees.

Thus, this study aims to map and analyze the risks and opportunities related to long-term equity investments for companies and governments engaged in an Olympic agenda. To this end, over the years, the literature has discussed the themes of direct investment and the Olympic Games in different realities. The articles of greater relevance can be categorized into three subgroups: those that took into account the impact of the Olympic Games on foreign issues (17, 31, 32); the event's impact on domestic issues (2, 16, 27); and the impact of the event on the destination image of the hosting regions (1, 4, 48). With these subgroups, it was possible to identify some challenges and opportunities related to investment in mega-event contexts.

The literature shows that mega-events differ in terms of the origin of the investments, which could be of public, domestic private, or foreign private origin (35). These investments are then responsible for financing a mega-event and stimulating the local business environment in this context (8). In addition to sporting mega-events, the focus of this work, it was also possible to identify other types of events responsible for affecting local businesses, namely, cultural, sectorial corporate, political, and economic events. In this sense, one of the Brazilian interviewees remarked, “After the Olympics, we need to rely on these other types of events to justify the investments made in previous years. Without them, the infrastructure and resources allocated could go underutilized.” Major pressures (challenges) for the realization and success of these events, some main variables were mapped, from which we highlighted issues that considered the pressures of the media, international political pressures, public security, health security, migration, human rights, and political freedom. Some of these, for example, were very evident in the 2016 Rio project, such as the public safety problems (49). One of the Brazilian interviewees stated that “From the beginning, one of the IOC's biggest concerns was how the city of Rio would be able to deal with the feeling of insecurity perceived by the international public,” and there were health security challenges posed by the COVID-19 pandemic in the Tokyo case between 2020 and 2021 (47).

The literature also presents other main legacies of the event, which could be positive or negative, tangible or intangible (9, 10). The impacts on the image of the destination areas, urban transformations of the hosting regions, public policies, economic and social development, internationalization of companies, and national and international tourism were identified. In line with this, one of the Brazilian interviewees noted, “Hopefully the legacy left by the Olympics will be capable of transforming the image of the city and foster international tourism in the coming years. It is a thing we need, but so far, still is not clear if will going to happen.” In addition, the literature also indicates that such pressures are different depending on where the event is being held, with a clear distinction between developed and emerging regions, the latter of which in recent years have received increasing attention for mega-events (50). As these countries commonly have different institutional structures (51), what was observed was the creation of similar problems and potentialities for each of the groups (52). “Since the 2000s, with the Sydney games in Australia, more and more countries in the southern hemisphere have been destinations for mega-events. How long this phenomenon will last, we don't know yet,” said one of the respondents interviewed for the Japanese case.

Due to the transformations that took place in the city of Rio de Janeiro that were mainly motivated by the 2016 Olympic Games, which deals with the tourist legacy left by the event (2), we can highlight that mega-events end up having a substantial impact on international investment flows, with a greater incidence in specific sectors of the economy, such as tourism, hospitality, urban mobility, and infrastructure (53). In the Rio case, it was possible to identify at least 8 billion dollars in investments in the aforementioned sectors (54), which changed the local competitive dynamics of entire industries over a period of just 5 years. One of the interviewees in the Brazilian case stated, “These investments have transformed the landscape of the city. It became evident all the structural transformations that Rio underwent; at one point, there was so much money that we didn't even know what to do with it.” In that context, different national and foreign companies expanded their business in the city in the years that preceded the 2016 Games, as mentioned by one of the interviewees who said: “We believed that the 2016 Olympic Games would inaugurate a new phase for the city, especially for the services sector. For this reason, no efforts and investments were spared to make us more competitive.”

Due to the crisis caused by the explosion in the offer of tourist-related services, together with the economic downturn and political crisis that hit the region in the period following the Games, the Rio case provides an understanding of the dynamics of competitiveness in the sectors most affected by the Games (55). In the case of Rio, it was possible to observe new competitive entrants who, due to the need for diversification in the period, began to compete beyond their traditional competitors. As a result, it was possible to observe the presence of new players such as Airbnb (56) putting additional pressure on the tourism industry's local competition. In this sense, one of the respondents stated: “When the Rio de Janeiro Olympic Games were planned back in 2009, multisided platforms were almost non-existent. But on the eve of 2016, we saw Airbnb and other platforms greatly increase the offer of services throughout the city.”

As a result of this diversification, necessary for the survival of local companies in the new context, together with the expansion of competitors, there was also an increase in possibilities both in terms of types of customers and sources of revenue. One of the interviewees observed: “We had to adapt to survive. Our focus on event tourism brings both opportunities and challenges. New revenue possibilities have emerged beyond traditional lodgings, and we are trying to focus on that.” To exert pressure on this dynamic, major challenges were identified, such as public safety, political and institutional instability, and the perceptions of the media and those in other national and foreign destinations can be highlighted, as noted by one of the interviewees, “Everyone, including the IOC, was afraid of the problems that public security could bring to the Games. Furthermore, the high bureaucracy and corruption history brought additional pressure on the delivery of the Games on time.” In this context, another important subject to mention is the acceleration of digital transformation in the world (57), especially since 2020 due to the COVID-19 pandemic. Together with this, it was also possible to see the advancement of these technologies that proved to be a real alternative to how companies do business or even how spectators consume sports and entertainment (58). These are changes that, in the long term, also tend to affect the dynamics of tourism in general (59). In addition to this, it was possible to identify the presence of the government and sectorial entities responsible for regulating and influencing these flows, both positively and negatively, (60).

Alongside the findings from these case studies, empirical evidence demonstrates that the institutional environment of a country or region directly influences a company's investment options, and incentives or constraints that are driven by the context of mega-events affect the creation of a favorable institutional environment (52). However, there is difficulty in identifying an investment’s origin (61), especially those that were made in the local companies, as, despite the presence of national and foreign brands, these brands do not always hold ownership of the operation, which often belongs to private investors. Thus, it was not possible to state that the perceived political risk may vary according to the company's trajectory and its national or foreign origin, as proposed by Kobrin (62). The planning problems observed in the case of Rio and other international mega sporting events of the period meant that over the years the relationship between the cost and benefits (legacies) of the event for countries and companies meant that they became increasingly disincentivized to invest in an Olympic agenda (31).

Thus, the Tokyo case was the first one capable of reflecting on how financial sustainability has been incorporated into the Olympic projects since the publication of the Olympic Agenda 2020 in 2014, and more recently the Olympic Agenda 2020 + 5 in 2021. Beyond that, the 2020 Tokyo Olympics still had to overcome additional challenges due to the COVID-19 pandemic, which led to the first postponement in Olympic history and a closed-door Games in the following year (63). “The postponement of the Olympic games was unprecedented. No one knew how to deal with that because it was a scenario never foreseen before”, said one of the respondents. Another one concluded, “Not even the worst critics could have imagined this scenario. Much is said about scenario planning methodologies in strategy, but no one predicted this one, even though a pandemic is not a new thing in history.”

The mapped threats and trends were responsible for exerting positive and negative pressures that were very present in Tokyo 2020 and may also serve as a point of attention for the next Olympic editions. Such dynamics can be better viewed in Figure 1, which helps clarify how the financial sustainability theme has been incorporated into the new Olympic agenda, especially since the impact of the pandemic on the Tokyo 2020 Games. “The sustainability of the Olympic Games was already something discussed in the Tokyo 2020 project. With the postponement and rising costs brought by it, reducing the impact of the Games has become even more essential,” said one of the interviewees.

It is important to mention that some of these threats were already present in previous articles, namely, public security (53, 64), institutional political security (65), and even health security (66), which in the context of a global pandemic were very present in the Tokyo Games and ended up gaining even more importance. Other variables such as the gigantism of the Games (15, 16), issues such as natural disasters and global warming (67), and information technology security (68) due to the acceleration of digital transformation in the world and in the Games itself, were very evident in the Japanese case. “Tokyo had already been heavily investing in technology even before the pandemic was known. Perhaps this strategy saved the organization from an even greater loss,” said one of the interviewees.

However, to mitigate part of these threats, other trends emerged, such as in the case of the projects that increased sustainability (69), which involves better management of the Olympic legacy (9, 27), and the rationalization of the infrastructure costs (70), to break with the gigantism of previous editions (71), as stated by one of the interviewees: “Initially, there was this idea of holding more responsible and sustainable games in terms of structures. The logic of reusing the 1964 stadium fits into that.” To propose an alternative to the high costs and risks involved in an Olympic project, the possibility of greater geographic permeability (72), i.e., shared candidacies between different cities and countries, is a trend for the future not only of the Games but also for other international mega sporting events. “Despite being called the Tokyo Games, we have competitions happening all around Japan. Baseball, soccer, and aquatic sports, for example, are not being held in the city. For the future, I believe we will see even greater geographic permeability, with the Olympics being hosted by different cities or even countries. The FIFA World Cup has already started this movement,” one of the interviewees added. Finally, we also had the impact of technology on sports, which influences not only the way the event is delivered but also how to interact with the public (57).

Many of these trends that had begun since the publication of the IOC's Agenda 2020 in 2014 are part of a greater worldwide transformation as they also demonstrate alignment with the United Nations’ sustainable development goals for 2030 (73). Such discussions have even more relevance in a world that for a few years will still carry the impacts of the political and economic crisis left by COVID-19, which ended up restricting, even more, the resources for large-scale projects such as the Olympic Games, which always had a difficulty in raising funds (26, 74). The complete dynamics can be seen in Figure 1.

Due to the preparation time required to host an Olympics, and its cyclical characteristic of occurring every 2 years if we consider the historical alternation that has been taking place between the Summer and Winter Games since 1924, there will always be governments and companies from somewhere in the world investing in an Olympic agenda (22). Thus, many possibilities emerge for the Olympic cities of the future to rethink their projects, precisely because of the possibility of observing the different variables provided by this work—including the risks to be avoided and opportunities to be maximized (Figure 1)—which deserves special attention in the event planning phase.

With the editions of mega sporting events that took place in the “Southern Hemisphere” (75), especially from the beginning of the 21st century, we may continue to observe this alternation between north and south and more or less developed host countries, and, together with that, all the challenges imposed by the characteristics of the institutional structures in each of these regions (76). “The door was opened in the 2000s with Sydney, and in the future, we should see even more emerging regions hosting mega-events, not just sports ones. Middle Eastern countries, for example, have been adopting this strategy for some years now as a way to transform their local image,” said one of the interviewees in the Tokyo case. Whether for Paris (2024), Milan (2026), Los Angeles (2028), Brisbane (2032), or the other cities that will come after them, the many challenges that are posed are mainly due to the increase in operational and financial complexity that the Olympic Games have experienced in their last editions (36, 77).

For Paris 2024, the challenge of balancing financial and operational sustainability for the Olympic Games was significant (78). According to Geffroy et al. (79), in Paris 2024, there was already a strong focus on reusing existing infrastructure and decentralizing events to other parts of France in an attempt to mitigate costs and maximize the benefits for various regions of the country. This strategic approach reflected an understanding that, to ensure the success of future games, an increasingly inclusive and resilient governance model will be necessary that is capable of accommodating the rising complexities of mega-events. The lessons learned in Rio, Tokyo, Paris, and other previous editions will be crucial in guiding future Olympic cities, which will also face similar challenges in terms of logistics, financing, and sustainable legacy. In support of this and according to one of the interviewees, “The issue of sustainability in the Olympic Games is an irreversible movement, which has been signaled by the IOC and societies in general for at least more than a decade.”

In contrast, despite the official discourse, what is often seen in practice is the opposite. The growth in sports modalities and international spectators has caused increasing demands for cities and companies searching for positive legacies, whether for their businesses or their destination image. However, the need for infrastructure and the ever-larger investments that have been required each time, in addition to the difficulty of financing, have resulted in the disapproval of societies that are more critical of the real benefits of hosting a mega-event (80, 81). “The increase in the number of so-called exhibition sports like surfing and skateboarding is an attempt to make the games more popular, especially among younger generations. However, it also generates the need for an even greater number of structures and investments,” stated one of the interviewees. Are these benefits justifiable to the point of the country giving up on other internal social agendas? The reality is that events that were once the most desired projects in the world have recently suffered from a growing disinterest due to their capacity to create more negative than positive legacies (33, 63).

This new dynamic that has taken over the Olympic agenda in recent years may in part justify cities such as Brisbane as the host of an Olympics, the awarding of which was held after an unprecedented single application. It is important to mention that this cannot be understood as an isolated event, since in the BID for the 2024 Games, there was a massive withdrawal of candidacies due to internal social pressures as in the case of Boston, Budapest, Hamburg, and Rome. With no candidates for the 2028 Games, the solution found by the IOC was to distribute the next two editions among the only two remaining candidates, Paris and Los Angeles. “An emerging country can be understood for its motivations to host an event like this, but for already developed regions, the current model of the Olympic Games is becoming increasingly unappealing, and this became evident by the recent withdrawals of candidacies,” said one of the interviewees. This trend highlights the increasing challenges and complexities that cities face in hosting the Olympics, prompting a reevaluation of the benefits and burdens associated with such a significant global event (82).

It is also important to remember that episodes like this have not been restricted to the Summer Olympic Games, and are also beginning to threaten the winter editions and other mega sporting events (83). Due to the unsustainability of the model in the medium and long term, the publication of the 2020 and 2020 + 5 Olympic Agendas by the IOC in 2014 and 2021, respectively, can be understood as a signal to future organizers and interested parties to change the direction of the Olympic movement toward a more inclusive, digital, and sustainable event (23). Despite the shift in the narrative, the practical implications of the new agenda guidelines were still difficult to see in the Tokyo case—partly because of the additional challenges imposed by COVID-19. “The publication of the Agendas by the IOC is a signal to countries and societies around the world that something needs to change in terms of Olympic model. Even so, this is a late reaction, and this transformation should have started a long time ago,” commented one of the interviewees.

In this sense, the model (Figure 1) can be understood as an embracing framework that was concerned with mapping the main flows and variables that affect investments in the context of the Games, through the identification of challenges and possible legacies of the dynamic, as well as focusing on how these markets react to main legacies left by the event. In this sense, special attention needs to be taken with regard to specific industries such as tourism and hospitality (53). The industries that are commonly the most affected by the Olympic investments need to pay attention to the legacies column of the model, but without forgetting the variables mapped in the group of main challenges to the Olympic Games as in the case of public health, which was very evident at the Tokyo case. It is worth mentioning that this variable was identified even before the crisis due to the COVID-19 pandemic, demonstrating to us that issues related to the health and safety of the event have always been present throughout its history (84).

Thus, the proposed model (Figure 1) not only aims to verify the impacts on the studied editions but also to identify the challenges that may be faced by future Olympic cities. These cities, in one way or another, will be affected by the initiated transformations that try to redefine the Olympic Games, by the understanding of the long-term effects of the Games, and by the necessary considerations for more sustainable and inclusive Olympic planning.

## 5 Final considerations

It is important to draw attention to the conjunctural events of recent years that were decisive in contributing to the acceleration of structural changes in the Olympic movement and directly responsible for how investments in the context of the Games were thought of. In this new moment, it is even more necessary to create a conscience in governments and companies on the issue of sustainability of the projects (85), which, mainly due to gigantism, has been one of the main barriers to Olympic candidacy. In a world in crisis due to war and economic constraints, especially after the COVID-19 pandemic that had political, economic, and social consequences in all nations, the availability of resources from countries and companies for the next Olympic editions tends to be even more restricted. Thus, one of the biggest challenges for future organizers will be to transform long-term intangible legacies into something more tangible and with short-term results, in a way that allows a better perception in the hosting regions of the economic sustainability of the Olympic projects (40).

The sustainability of future projects requires making expenses more rational (70), using existing infrastructures or even shared applications between different cities and countries, and thus providing other directions than the course to gigantism (15, 16). Alongside this, better legacy management (27, 35) becomes one of the only ways to justify the billions of dollars invested in the Olympic agendas. Non-existent or even negative legacies of past editions have also been responsible for putting off candidates (11). In addition, we draw attention to the digital transformation that took place not only in sports but also around the world (86). Due to the context of social isolation brought about by the COVID-19 pandemic, there was an acceleration of this phenomenon, which gradually turned from a tendency into a need. Thus, the sustainable development agenda has increasingly considered the capacity of events and businesses to replace the physical with the digital, through the concept of digital sustainability (87).

In addition, it is important to maintain the relevance of the discussion involving emerging and developed regions, since, depending on where the event happens, risks and opportunities commonly characteristic of these groups of countries tend to be maximized (88). Recent examples have shown that the alternation between the north and south hemispheres, especially since the Sydney Games in 2000, despite opening possibilities for a new class of countries and cities to host the Olympics, increases the risk of delivery and the legacy left by the event, especially due to the less developed institutional environment that is usually found in these regions, as we have seen in the recent Olympic examples of Athens (2004), Beijing (2008) and Rio de Janeiro (2016), and South Africa (2010), Brazil (2014), and Russia (2018) in the context of the FIFA World Cup.

Since developed and emerging countries have historically had different institutional structures (89), with their advantages and disadvantages, it is also necessary to reflect on the institutional variable (76) as something that determines the long-term sustainability of firms’ investments, since the favorable environment created by incentive policies and the market speculation brought by the events tend to be temporary, while direct investments would need more solid and long-term foundations.

It is also essential to recognize the roles of scholars, practitioners, and policymakers in addressing these challenges. Scholars should investigate innovative approaches to enhance the sustainability of the Olympic Games, considering multidimensional aspects such as environmental, economic, and social factors (5). In addition, they should explore the effectiveness of alternative legacy management practices (9) and how shared hosting can mitigate exorbitant costs and risks (15). Practitioners, including event organizers and business managers willing to invest in an Olympic context, need to adopt more efficient expenditure strategies and leverage existing infrastructures to ensure the long-term viability of investments (10). Finally, policymakers must develop and enforce regulations that foster sustainability and provide stable, long-term institutional frameworks (76). They should also consider the unique challenges of hosting mega-events in both emerging and developed regions (89), ensuring tailored support to maximize positive outcomes and minimize risks (13).

In this context, five suggestions for future research can be proposed, namely, (1) explore the sustainability of the Olympic Games in its broadest sense, including not only environmental issues but also the economic and social aspects; (2) understand the importance and how the rationalization of Olympic project expenditure will impact the sustainability of the Olympic cycles in the medium and long term; (3) investigate alternative legacy management methodologies to justify the high Olympic investment, balancing the benefits brought by the event; (4) analyze the possibilities of combined Olympic candidacies as a way to make the event more accessible, dividing costs and risks between locations; and (5) understand how the worldwide digital transformation with the intensive use of technology in sport will affect the model of the Olympic Games and other mega sporting events in the future.

In terms of the limitations of this study, one is its reliance on qualitative methodologies, which, while providing in-depth insights, may not capture the full complexity of the Olympic investment landscape. The sample size, consisting of in-depth interviews of only the Rio 2016 and Tokyo 2020 cases, may limit the generalizability of the findings to other Olympic Games or mega-events. In addition, the study's focus on only two Summer Olympics, which occurred under unique circumstances—such as Rio's political and institutional instability and Tokyo's hosting during a global pandemic—might not fully represent the broader range of challenges and opportunities faced by different host cities. Furthermore, there is an inherent difficulty in quantifying and evaluating the long-term impacts of Olympic investments, particularly in terms of intangible legacies and sustainability (27).

Future research should consider a larger and more diverse sample, employ mixed-method approaches, and extend the analysis to other types of mega-events to enhance the robustness and applicability of the findings. There is also a significant opportunity to conduct quantitative studies on the topic, which could provide more objective and statistically significant insights into the economic, social, and environmental impacts of Olympic investments. By employing quantitative methodologies, researchers can better assess the extent of the benefits and risks associated with hosting the Olympics, ultimately contributing to a more comprehensive understanding of how to optimize investment strategies and ensure sustainable outcomes for future Olympic Games. Moreover, it is crucial to study other Olympic cases and other mega-events to understand how contemporary challenges and evolving contexts impact investment dynamics and legacy outcomes. Such studies would provide updated insights that reflect current trends and innovations in event hosting, offering valuable guidance for future hosts and stakeholders.

## Data Availability

The original contributions presented in the study are included in the article/Supplementary Material, further inquiries can be directed to the corresponding author.

## Footnotes

^1^The Olympic BID is the process of choosing the next Olympic city. The entire process usually takes 2 years to conclude and happens 9 years before the Olympic edition in question.
